# OMRT-1. Cannabidiol converts NFKB into a tumor-suppressor in glioblastoma with defined antioxidative properties

**DOI:** 10.1093/noajnl/vdab070.027

**Published:** 2021-07-05

**Authors:** Marie Volmar, Jiying Cheng, Michael Synowitz, Joel Schick, Roland Kälin, Rainer Glass

**Affiliations:** 1 University Clinics Munich, Munich, Bavaria, Germany; 2 University Clinics Kiel, Kiel, Schleswig-Holstein, Germany; 3 Helmholtz Center Munich, Munich, Bavaria, Germany

## Abstract

**Background:**

The transcription factor NFKB drives neoplastic progression of many cancers including primary brain tumors (glioblastoma; GBM). Precise therapeutic modulation of NFKB-activity can suppress central oncogenic signalling pathways in GBM, but clinically applicable compounds to achieve this goal have remained elusive.

**Methods:**

In a pharmacogenomics study with a panel of transgenic glioma cells we observed that NFKB can be converted into a tumor-suppressor by the non-psychotropic cannabinoid Cannabidiol (CBD). Subsequently, we investigated the anti-tumor effects of CBD, which is used as an anticonvulsive drug (Epidiolex) in pediatric neurology, in a larger set of human primary GBM stem-like cells (hGSC). For this study we performed pharmacological assays, gene-expression profiling, biochemical and cell-biological experiments. We validated our findings using orthotopic in vivo models and bioinformatics-analysis of human GBM-datasets.

**Results:**

We found that CBD promotes DNA-binding of the NFKB-subunit RELA and simultaneously prevents RELA-phosphorylation on serine-311, a key residue which permits genetic transactivation. Strikingly, sustained DNA-binding by RELA lacking phospho-serine 311 was found to mediate hGSC-cytotoxicity. Widespread sensitivity to CBD was observed in a cohort of hGSC defined by low levels of reactive oxygen-species (ROS), while high ROS-content in other tumors blocked CBD induced hGSC-death. Consequently, ROS-levels served as predictive biomarker for CBD-sensitive tumors.

**Conclusions:**

This evidence demonstrates how a clinically approved drug can convert NFKB into a tumor-suppressor and suggests a promising repurposing option for GBM-therapy.

